# Microplastic Passage through the Fish and Crayfish Digestive Tract Alters Particle Surface Properties

**DOI:** 10.1021/acs.est.4c08909

**Published:** 2025-03-14

**Authors:** Ewa Babkiewicz, Julita Nowakowska, Marcin L. Zebrowski, Selvaraj Kunijappan, Katarzyna Jarosińska, Rafał Maciaszek, Jacek Zebrowski, Krzysztof Jurek, Piotr Maszczyk

**Affiliations:** † Department of Hydrobiology, Institute of Ecology, Faculty of Biology, University of Warsaw, Warsaw 00-927, Poland; ‡ Biological and Chemical Research Centre, University of Warsaw, Warsaw 02-089, Poland; § Imaging Laboratory, Faculty of Biology, University of Warsaw, Warsaw 00-927, Poland; ∥ Department of Biotechnology, Kalasalingam Academy of Research and Education, Krishnankoil 626126, India; ⊥ Warsaw University of Life Sciences, Institute of Animal Science, Department of Animal Genetics and Conservation, Warsaw 02-787, Poland; # Institute of Biotechnology, College of Natural Sciences, University of Rzeszow, Rzeszow 35-310, Poland; ∇ Faculty of Geology, Geophysics and Environmental Protection at the AGH University of Krakow, Kraków 30-059, Poland

**Keywords:** microplastics, microstructure, scanning electron microscopy, ATR-FTIR, Py-GC/MS, ingestion, biodegradation, bacteria, fish, crayfish, digestive tract

## Abstract

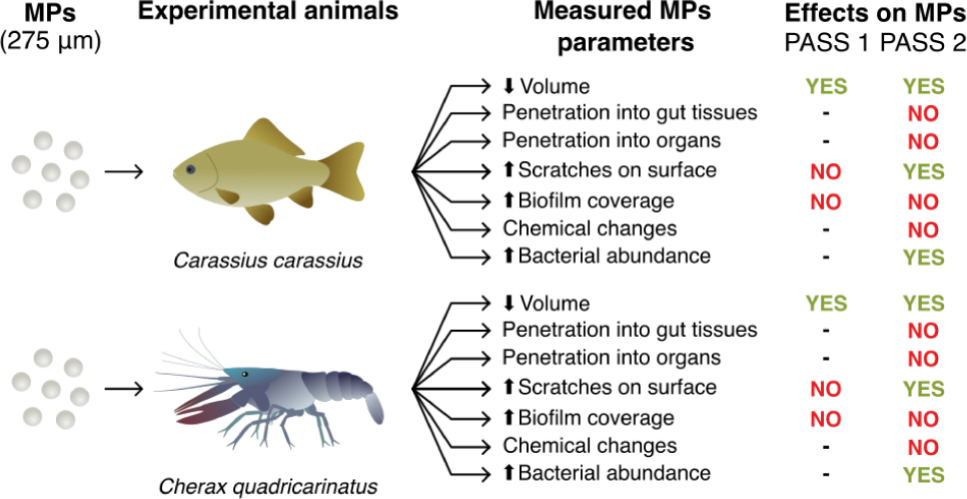

Most studies on the effects of organisms on microplastic characteristics have focused on microorganisms, while the impact of animal feeding behavior, particularly in aquatic species like fish and decapod crustaceans, has been less explored. This study examines how polyethylene spherical microplastics (275 μm in diameter) passing through the digestive tracts of crucian carp (*Carassius carassius*) and Australian crayfish (*Cherax quadricarinatus*) affect surface properties, particle size, and bacterial colonization. The species were fed diets with or without microplastics. The particles underwent two rounds of passage through the digestive tracts and were then exposed to known bacterial densities. Surface damage, size, and biofilm coverage were analyzed using scanning electron microscopy, while alterations in surface chemical composition were assessed through Fourier transform infrared spectroscopy with attenuated total reflectance, and the formation and penetration of nanoplastics in gut tissues and glands were determined using Py-GC/MS. Results show that the passage significantly altered surface properties and reduced microplastic size, without affecting chemical composition or nanoplastic penetration into tissues. These changes promoted bacterial colonization compared to controls. The findings suggest that animal feeding activity may play an important role in the mechanical fragmentation of microplastics in aquatic environments, potentially leading to their faster degradation.

## Abstract 2

This study examines how the feeding activity of fish and crayfish alters the surface properties of microplastics, enhancing bacterial colonization and potentially influencing microplastic degradation in aquatic environments.

## 1 Introduction

Plastics represent a heterogeneous group of materials comprising various synthetic or semisynthetic organic compounds, often in the form of large polymers (e.g., polyethylene (PE), polystyrene (PS), polypropylene (PP)). Over the past several decades, global production and utilization of plastics have increased, raising concerns about their accumulation in the environment [e.g.,^1^] In recent years, increasing attention has been directed toward the proliferation and potential environmental impacts of microplastics (MPs), typically defined as plastic particles within the size range of 1 μm to 5 mm in their largest dimension.^2^ Particles smaller than 1 μm are classified as nanoplastics (NPs).^2^ These particles result from the disintegration of larger plastic fragments and the release of plastic particles already produced in small sizes, such as those found in personal care products.

Many organisms in natural environments ingest MPs [e.g.,^3−5^] The harmful effects of ingested MPs have been documented across all levels of biological organization [e.g.,^6−10^] The extent of harm may depend on the specific characteristics of the particles, including their type, size, density, and even color [e.g.,^11^]

Despite extensive research, the actual quantity of MPs present in the environment remains poorly understood and is often lower than previously estimated.^12^ One potential explanation for this discrepancy is the underestimation of the impact of degradation processes. The degradation of plastics, including MPs, is a process that results in alterations to the chemical, mechanical, electrical, and optical properties of polymers due to chemical, physical, and biological processes [e.g.,^13^] This degradation leads to the breaking of bonds and subsequent transformations of the material.^14^ While several studies define degradation as resulting in the complete mineralization of the polymer, most also acknowledge the incomplete degradation of particles, which includes physical fragmentation, such as surface scratches on plastics and the detachment of fragments.^15^ The degradation of polymers can be influenced by a variety of factors, both abiotic and biotic. Abiotic factors encompass a range of processes, including photo-oxidative, thermal, ozone-induced, mechanical, and catalytic degradation. Biotic degradation includes both physical damage to particles (such as biting, chewing, and digestive fragmentation) and biochemical processes.^12,16,17^

Research on the effects of organisms on the degradation of MPs has primarily focused on microorganisms such as fungi [e.g.,^18,19^] bacteria [e.g.,^20,21^] and unicellular algae.^22^ However, there is a lack of information regarding the influence of animals, particularly vertebrates, on the qualitative characteristics of MPs.^17^ Only four studies have provided evidence of MPs degradation, mainly fragmentation, by aquatic invertebrates.^23−26^ For example, it has been demonstrated that Atlantic krill (*Euphausia superba*) can fragment PE-MPs into NPs within 4 days of digestion, thereby facilitating their penetration into gut tissues.^23^ However, there is no evidence in the literature to suggest that crayfish and fish activity affects the degradation of MPs, despite their pivotal role in the functioning of most aquatic environments.

It has been demonstrated that decapods and fishes are capable of ingesting MPs [e.g.,^4,27−29^] Research on invertebrates indicates that the primary degradation pathway of MPs likely occurs through their digestive systems.^17^ As MPs move through the digestive system, they undergo various physical and chemical processes that can alter their characteristics. These processes may include mechanical grinding, exposure to digestive enzymes, and interactions with the gut microbiome, potentially leading to changes in the number, size, shape, or surface properties of MPs, including increased scratching and biofilm coverage.^17^

Mechanical processes in cyprinid fish may involve friction from their pharyngeal teeth, allowing them to ingest both food and inedible particles^30−32^ potentially even crushing plastics. In crayfish, mechanical processes such as grinding in the stomach^33^ and abrasion from gastroliths—small stones that are swallowed or produced internally^34^—can contribute to breaking down materials. Additionally, damage can occur during food manipulation by crayfish mouthparts. Biochemical processes may also degrade MPs, as gastric enzymes can cause hydrolytic breakdown.^25^ Bacterial activity in the digestive systems of animals may further break down MP surfaces through enzyme production.^20,35^ Both crucian carp and freshwater crayfish have diverse digestive microbiota^36−38^ that could enhance MP degradation. Even if MPs pass through the digestive tract too quickly for complete breakdown, this process might initiate surface modifications, promote microbial colonization, and create a chemically favorable environment for bacteria. Degradation could intensify with repeated passages through the digestive systems of the same or different animals.

The objective of this study is to test five hypotheses regarding the effects of PE-MPs passing through the digestive tracts of crucian carp (*Carassius carassius*) and Australian redclaw crayfish (*Cherax quadricarinatus*): (1) Mechanical processes in the digestive tracts of these animals cause physical surface alterations to PE-MPs and generate fragments that may penetrate gut tissues and glands; (2) Digestive conditions promote biofilm formation on PE-MPs; (3) Chemical interactions in the digestive tracts alter the surface properties of PE-MPs; (4) Structural modifications from digestive processes facilitate bacterial colonization of PE-MPs; and (5) Repeated passage through digestive tracts amplifies physical damage and bacterial colonization on PE-MP surfaces.

## 2 Material and Methods

### 2.1 Experimental Animals

Twelve juvenile crucian carp (*C. carassius*; body length: 8.0 ± 1.0 cm; fresh weight: 15.0 ± 2.3 g) of both sexes were used in the first main experiment, with an additional two individuals used in a supplementary experiment. The fish were sourced as juveniles from the “Fish for Ponds” facility in Grajewo, Poland, where they were hatched in 2023. They were maintained at 21 °C with a 16L:8D photoperiod in 100 L glass tanks filled with aerated tap water connected to a flow-through biological purification system. Twelve juvenile Australian redclaw crayfish (*C. quadricarinatus*; body length: 5.0 ± 0.6 cm; fresh weight: 8.0 ± 1.1 g) of both sexes were used in the second main experiment, with an additional two individuals used in a supplementary experiment. The crayfish were sourced from the “Kumak Shrimp” breeding facility in Konstancin-Jeziorna, Poland. Prior to the experiments, they were kept in a 30 L aquarium with filtered tap water, aeration, sand at the bottom, and coconut shells for shelter.

During the breeding process, both the fish and crayfish were fed small amounts of frozen Chironomidae larvae (Ichthyo Trophic, Poland). Seven days before the experiments, their diet was switched to groundbait (made from wheat flour and ethyl vanilla, Dragon, Poland) for 5 days. In the final 48 h before the experiments, the animals were not fed to cleanse their digestive tracts. During the experiments, both fish and crayfish were fed groundbait mixed with approximately 150 PE-MP particles.

The research was conducted with the approval of the Local Ethics Committee in Warsaw (Permit protocol No. 1350P1/2022).

### 2.2 Experimental System

The experimental setup for the main experiments involving both fish and crayfish included 20 glass aquaria (9 L each; 26 cm × 17 cm × 20 cm), each filled with 7 L of aerated and conditioned tap water. The same setup was used for both experiments, but the experiments were conducted at different times, with the crayfish experiment conducted first. The aquaria were maintained in a laboratory room at a stable temperature of 21 °C ± 0.2 °C with consistent lighting conditions. Light intensity was set to 10.0 ± 0.5 μmol × m^–2^ × s^–1^ just below the water surface, measured with a Li-Cor 189 quantum sensor, to mimic a summer photoperiod (16L:8D). Temperature and oxygen levels were monitored using a YSI ProODO oxygen probe. In the experiment with fish, 12 aquaria housed a single fish each. The remaining 8 aquaria served as controls, with 4 allocated to the variant containing MPs in the water and 4 to the variant with MPs mixed with groundbait. Both experimental and control aquaria were arranged randomly. The same setup was used in the experiment with crayfish, with the only exception being that a coconut shell was placed in each aquarium with an animal to serve as a shelter. The shell was also placed in each of the control aquaria.

The setup for the supplementary experiment was the same as in the main experiment, with one difference: it consisted of 4 aquaria instead of 20. Two aquaria housed a single fish each, while the other two housed a single crayfish each. In both pairs, one individual corresponded to the control variant (fed groundbait without MPs), and the other to the experimental variant (fed groundbait with MPs).

### 2.3 Microplastics Used

A conventional MPs in the form of spherical, milky-white microsphers made of high-density polyethylene (LDPE) were used with density = 0.96 g × cm^3^ and a mean diameter of 275 ± 25 μm (cat. no. CPMS-0.96, Cospheric, UK). LDPE is commonly recognized as a nonbiodegradable polymer. It exhibits a hardness of approximately 32.4 Hs on the Shore D scale and an impact energy absorption of 12.68 J × m^–1^.^39^ The number-average molecular weight (Mn) of the LDPE used is approximately 30 000 g/mol, with a weight-average molecular weight (Mw) of 250 000 g× mol^–1^. This reflects the presence of long-chain molecules with varying lengths due to its branched structure.^40^ The molecular number is approximately 1.92 × 10^19^ molecules per cm^3^. The purity of the CPMS-0.96 product exceeds 99.9%, as confirmed by the manufacturer, indicating the absence of additives.

### 2.4 Procedure for the Two Main Experiments

Two 12-day experiments were conducted, each consisting of 12 replicate aquaria (each housing one animal) and 8 control aquaria (4 with MPs in water and 4 with MPs in food). Each experiment was divided into two six-day stages, representing two gastrointestinal passages of MPs. The first experiment involved crayfish, while the second focused on fish. Prior to the experiments, all aquaria were filled with filtered and aerated tap water (filtered using a 1 μm polypropylene fiber filter, FCPS1, AquaFilter, USA). Animals were then individually introduced into the experimental aquaria using a net made from natural materials to minimize contamination risks.

For the experimental procedure, loose groundbait was mixed with water and kneaded into an elastic mass. Approximately 150 PE-MP particles were added to the groundbait and thoroughly mixed in. Granules approximately 3–4 mm in diameter were formed to facilitate easy swallowing by the animals. After a 1-h acclimation period, the oxygen concentration (8.1 ± 0.5 mg O_2_ × L^–1^) and temperature (21.0 °C ± 0.2 °C) were checked. Each animal was fed a single small groundbait ball (∼3–4 mm in diameter, 150 mg) once per day. Similarly, one groundbait ball was added daily to the first type of control aquaria (control with MPs in food). Simultaneously, 300 MPs were introduced into the water of the second type of control aquaria (control with MPs in water). After 40 min, any uneaten groundbait and MPs released during feeding were removed by changing the water to conditioned water, temporarily placing the animal in a separate tank. During this water change, each aquarium (and the coconut shell in the case of crayfish) was rinsed several times to eliminate any remaining MPs.

After 24 h, all excreted feces were collected, and the postexperimental water was gently sieved through a 20 μm mesh to retrieve all MPs. This procedure was repeated for the remaining 5 days of the experiment. Every day, MPs collected from the experimental aquaria and corresponding control aquaria were pooled separately across the six feeding sessions and placed in a glass bottle dedicated to each aquarium. The collected MPs from all six experimental days were placed in a single container for each of the 20 aquaria (12 experimental and 8 control aquaria). The same procedure was followed for each aquarium, regardless of whether it was experimental or control, ensuring that samples were processed consistently.

Before the second passage, the MPs collected from the first passage (along with those from the corresponding controls) were separated from particulate matter by gentle sieving through a 20 μm mesh. Half of each sample was retained for analysis after the first passage, while the other half was used for the second passage. During the second passage, the entire procedure was repeated, except that the groundbait balls were mixed with MPs recovered from the first passage.

After each passage, the retrieved and precleaned particles were gently separated manually under a binocular microscope and then transferred to 15% hydrogen peroxide for 48 h to remove biofilm and any remaining groundbait. Prior to this, several particles from each sample were isolated for biofilm coverage analysis. The same procedure was applied to the control samples. Next, the particles were divided into three subsamples. Particles from the first subsample were dried at 40 °C (Salvislab Thermocenter TC100, Switzerland) for 24 h and then stored in a dry, dark, and cool environment until imaging with a scanning electron microscope (SEM) to assess mechanical abrasion. Particles from the second subsample were exposed to a medium containing bacteria from the aquarium with a single fish or crayfish placed in 9 L for 12 h, which was filtered through a 20 μm mesh. This medium corresponded to the experimental conditions and initially contained approximately 8.12 ± 2.42 × 10^6^ bacteria × ml^–1^. The number of bacteria was assessed using DAPI staining and fluorescence microscopy (2.7. DAPI Staining of Free-Living Bacteria). After 48 h, the particles were preserved with 0.2% formaldehyde and dried at 40 °C (Salvislab Thermocenter TC100, Switzerland) for 24 h. They were then kept in a dry, dark, and cool environment until imaging with a SEM to count the number of bacteria on the surface. Particles from the third subsample were also dried at 40 °C (Salvislab Thermocenter TC100, Switzerland) for 24 h and subsequently stored in a dry, dark, and cool environment to determinate chemical changes on the surface of the particles by Fourier transform infrared spectroscopy using attenuated total reflectance (ATR-FTIR, model IRSpirit-T, Shimadzu, Japan).

### 2.5 Procedure for the Supplementary Experiment

A two-day supplementary experiment was conducted using 2 fish and 2 crayfish housed in separate aquaria. In each pair, one individual was fed groundbait without MPs (control), and the other was fed groundbait containing MPs (experimental). On the first day, MPs passed through the digestive tract for the first time, and on the second day, for the second time.

Both control and experimental animals were fed in the same manner as in the main experiments, followed by a 24-h period to allow gut evacuation. After the second passage, all four animals were anesthetized using buffered MS-222 (tricaine-methanosulfonate, 400 mg × L^–1^). Intestines, livers, and hepatopancreases were collected, and 0.0044–0.127 g of each tissue sample was used for NPs extraction.

The extraction followed a five-step procedure based on.^41^ First, 1 mL of 10 M NaOH was added to tissue samples minced with metal scissors, and the mixture was shaken at 300 rpm for 24 h at room temperature to fully solubilize the tissues. The MP content was separated by filtration through a glass microfiber filter membrane (1 μm, Whatmann). To the filtrate, 10 mL of 99% ethanol (Chempur) was added, and the mixture was incubated in a water bath at 80 °C for 30 min. The solution was centrifuged at 2000 rpm for 5 min to precipitate protein-loaded NPs. The supernatant was removed, and trace protein and extracted NPs in the pellet were redispersed by adding two drops of ultrapure water.

The NP dispersion was then repeatedly transferred to quartz tubes (25 mm in length, 1.9 mm inner diameter), secured with quartz wool, and dried at 100 °C for 10 min to remove water. The prepared samples were used for subsequent Py-GC/MS analysis.

### 2.6 SEM Analysis of MPs Surface Parameters

**Figure 1:**
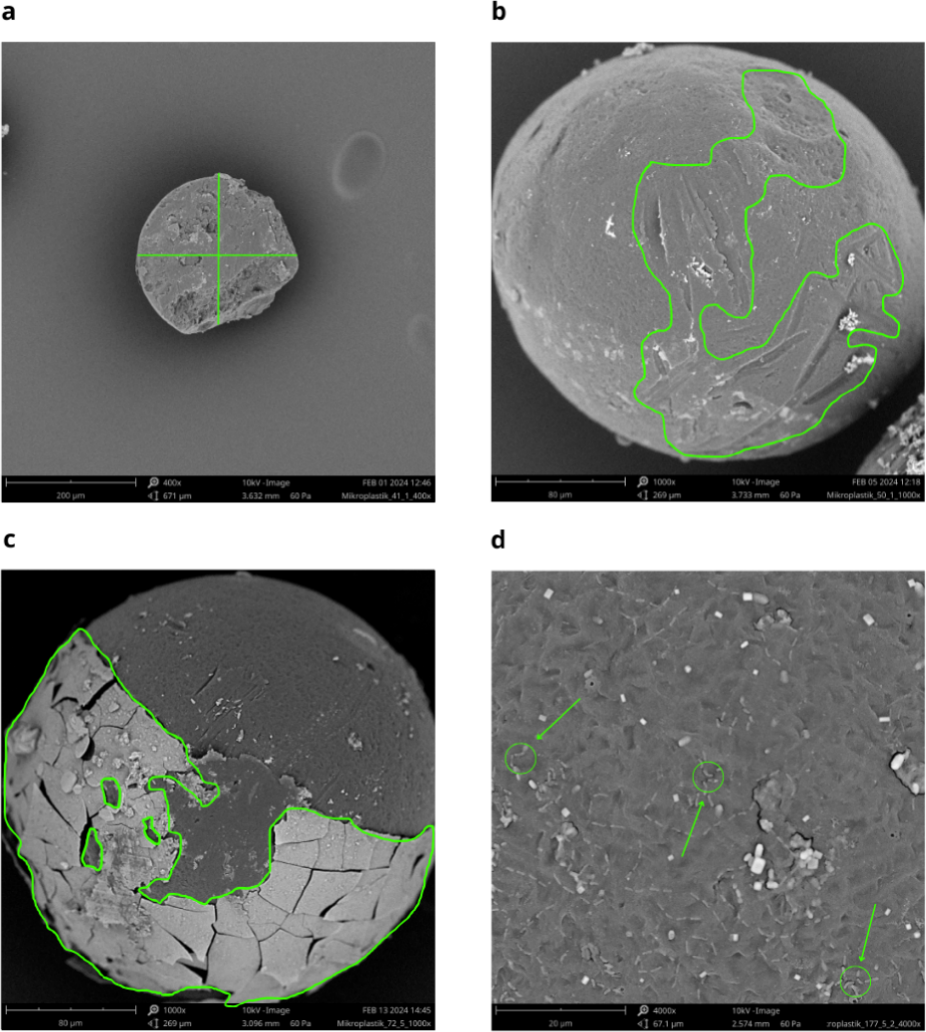
SEM images of representative MPs: (a) delineated diameters, magnification ×400; (b) delineated surface scratches, magnification ×1000; (c) particle surface covered by biofilm, cracked after the drying process, magnification ×1000; (d) bacterial aggregation, magnification ×4000.

The surface area of the MPs covered by scratches, particle diameter, biofilm coverage, and bacterial density on the surface (Figure 1) were analyzed using Scanning Electron Microscopy (SEM) imaging. MPs images were captured in the Imaging Laboratory at the Faculty of Biology, University of Warsaw. The dried MPs were mounted on a SEM stub and sputter-coated with gold using the POLARON SC7620 metal sputtering machine (Microtech). They were examined with the Phenom ProX (Phenom-Vorld BV**)** scanning electron microscope at various magnifications: 50× for general appearance, 400× for measuring diameters, 1000× for observing surface scratches and particle surfaces covered by biofilm that cracked after the drying process, and 4000× for bacterial aggregation (Figure 1).

### 2.7 DAPI Staining of Free-Living Bacteria

To determine the density of bacteria in water, we employed standard 4′,6-diamidino-2-phenylindole (DAPI) staining.^42^ For this purpose, we diluted 1 mL of water sample from the fish or crayfish aquarium in 9 mL of Milli-Q water and added 200 μL of 50 μg × ml^–1^ DAPI. The sample was then kept in darkness for 15 min. Next, we filtered the stained sample through a polycarbonate black membrane filter with 0.2 μm pores (Nucleopore). The density of bacteria (*Db*) was calculated using the formula: *Db* = (*nb* × *Nf*)/(*nf* × *V*) where *nb* is the total number of bacteria in the fields of view, *nf* is the number of fields of view counted (14), *Nf* is the total number of fields of view on the filter (the ratio of the total filter area to the field of view area = 43 388), and *V* is the volume of the subsample (1 mL).

### 2.8 ATR-FTIR Analysis of Chemical Changes in MPs

Chemical changes on the surface of the MPs were analyzed on individual MPs particles, collected from second passage, after intensive cleaning with a stream of deionized water, using FTIR spectroscopy. The measurements were performed with an IRSpirit-T (Shimadzu, Japan) spectrometer coupled with an ATR accessory (QATR-S) at a resolution of 4 cm^–1^ and 64 scans. Prior to each measurement, the surface of the diamond crystal was cleaned with 70% ethanol to remove residues from previous samples, and a new background was collected. The spectra were preprocessed using rubberband baseline correction and normalized to the highest peak, which corresponds to the CH_2_ asymmetric stretching vibrations.

### 2.9 Py-GC/MS Analysis

The analysis of PE-NPs was conducted at the Faculty of Geology, Geophysics, and Environmental Protection at the AGH University of Krakow. A Pyroprobe 5000 series was used for pyrolysis, integrated with an Agilent Technologies 7890A GC system. The system featured an Agilent J&W DB-5 ms Ultra Inert Column (30 m × 0.25 mm × 0.25 μm) and was coupled to a 5975C Inert MSD with a Triple-Axis Detector.

The pyrolysis and chromatographic methods followed those described by.^43^ Pyrolysis was performed online for 15 s at 590 °C, with helium as the carrier gas at a constant flow rate of 0.8 mL × min^–1^. The pyrolysis products were introduced into the GC system via a split/splitless inlet maintained at 300 °C, operating in split mode with a split ratio of 1:15.

The temperature program began at 50 °C, held for 1 min, and was then increased at a rate of 3 °C per minute to 310 °C, where it was maintained for 10 min. The mass scan range was set from 41 to 650 amu, with simultaneous registration of selected ions at masses 83 and 85. The limit of detection (LOD) for the analytical method was 0.5 μg of PE.

### 2.10 Data Analysis

To assess the ratio of altered to unaltered surfaces, the number of visible bacteria on the surface, and the presence or absence of biofilm in the SEM images, we utilized ImageJ 1.53g software.^44^

Statistical analysis was performed using R version 4.3.2 (R Core Team, 2023) with a significance level (*α*) of 0.05. Normality was assessed using Shapiro-Wilk tests, and homogeneity of variance was evaluated with Levene’s test.^45^ The R code for these tests, executed in RStudio, is provided in the Text S1.

The data were analyzed in two-factorial design. The first factor, MP treatment, had four levels: Control 1 (C1)—MPs in the substrate without bait, Control 2 (C2)—MPs in the substrate mixed with bait, Fish (F)—fish exposed to MPs, and Crayfish (CF)—crayfish exposed to MPs. The second factor, MP exposure (passage), had two levels: first and second passage. Both factors were treated as fixed effects.

The volume of MPs and the bacterial density on the surface of MPs were analyzed using general linear models (LMs). The bacterial density analysis, performed only for the second passage, used a one-factorial design. The percentage of MPs covered by scratches and their biofilm surface coverage were analyzed using generalized linear models (GLMs;^46^ with a beta distribution and a “logit” link function, implemented via the glmmTMB package (v.1.1.310;^47^ Model diagnostics were conducted using DHARMa scaled residual plots (DHARMa package v.0.4.56.^48^

To evaluate the significance of main effects and the interactions between factors the sum of squares, degrees of freedom, F-test and *p*-value were calculated for LMs using the Anova() function from the car package v.3.1–3.^49^ The same package was used for the analysis of deviance with Wald type II chi-square (χ^2^) tests for GLMs, evaluated using Fisher’s exact test (F-test) for LMs, and for (calculated using the Anova function from the car package v.3.0–12;^49^*Posthoc* comparisons employed planned contrasts for estimated marginal means (EMMs; emmeans package v.1.710.25;^50^ with Holm’s adjustment to control for type I error inflation. Spectral clustering was examined using principal component analysis (PCA), conducted with the factoextra package.^51^

## 3 Results

### 3.1 Volume of MPs

**Figure 2:**
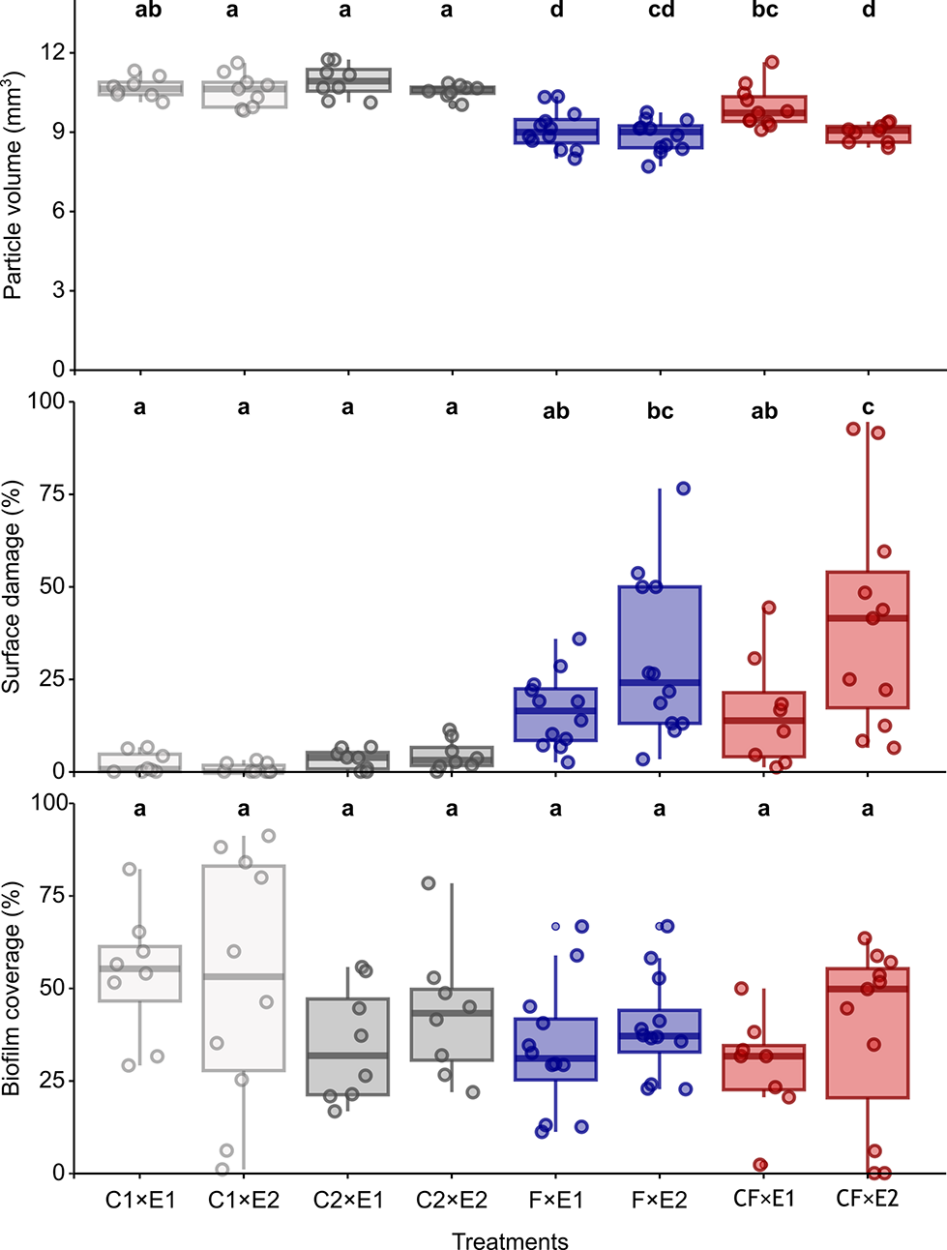
Volume, surface area of MPs and surface area of MPs covered by biofilm across different treatments: C1-E1 (control 1 after the first exposure of MPs), C1-E2 (control 1 after the second exposure of MPs), C2-E1 (control 2 after the first exposure of MPs), C2-E2 (control 2 after the second exposure of MPs), F1-E1 (after the first passage through the fish tract), F1-E2 (after the second passage through the fish tract), C1-E1 (after the first passage through the crayfish tract), and F1-E2 (after the second passage through the crayfish tract). The vertical line on the boxplots indicates the median, while the interquartile range represents 50% of the data. The top and bottom whiskers represent the 25th and 75th percentiles of the results, respectively. Significant differences between the investigated comparisons (Tables S2, S4 and S8) are denoted by different Latin letters.

The volume of MPs differed significantly between treatments and exposures (LM; *p* < 0.001 and *p* = 0.003, respectively; Table S1). However, the interaction between the two factors was not significant (LM; Table S1). Notably, the first passage of MPs through the digestive tract of the fish resulted in a significant reduction in MPs volume compared to both controls (*p* < 0.001 for both; Table S2, Figure 2). The second passage of MPs through the digestive tract of the crayfish also resulted in a significant reduction in MPs volume compared to control 2 only (planned contrast; *p* = 0.006; Table S2; Figure 2), although the difference for control 1 was very close to significance (planned contrast; *p* = 0.057; Table S2; Figure 2). Additionally, the volume of MPs after ingestion by fish was lower than that after digestion by crayfish (planned contrast; *p* = 0.011; Table S2; Figure 2). The volume of MPs was significantly lower after the second passage through the gastrointestinal tract of both fish and crayfish compared to both controls (planned contrast; *p* < 0.001; Table S2; Figure 2). The mean volume of MPs was lower after the second exposure compared to the first for both species, but significance was observed only after consumption by crayfish (planned contrast; *p* = 0.007; Table S2; Figure 2).

### 3.2 Surface Area of MPs Covered by Scratches

The surface area of MPs covered by scratches differed significantly between treatments and exposures (GLM; *p* < 0.001 and *p* = 0.019 respectively, Table S3). The interaction between these two factors was also significant (GLM; *p* = 0.045; Table S3). The surface area of MPs covered by scratches was not affected by the first passage through the digestive tract of either fish or crayfish (planned contrast; Table S4, Figure 2). After the second exposure, the scratched surface area was significantly larger after consumption of both fish and crayfish compared to both controls (planned contrast; *p* < 0.001 *p* = 0.040; Table S4; Figure 2). The only exception was the insignificant effect of the second passage of the fish tract compared to control 2. In addition, the surface area was larger after the second passage compared to the first, but only in the case of crayfish (planned contrasts; *p* = 0.008; Table S4, Figure 2).

### 3.3 Nanoplastic Presence in Animal Tissues

The characteristic sequence of triplets of PE pyrolysis products (Figure S1a) was not detected in any of the control or experimental samples analyzed (Figure S1b–e), indicating negligible penetration of PE-NPs into gut tissue and glands in both fish and crayfish. In certain pyrograms, such as the experimental sample from crayfish’s hepatopancreatic tissue (Figure S1b), peaks corresponding to *n*-alkenes were identified, while *n*-alkanes and *n*-alkadienes were absent among the pyrolysis products. The presence of *n*-alkenes alone may be attributed to the sample matrix, as observed in the control sample from pancreatic tissue (Figure S1c), rather than to the presence of PE.

### 3.4 Biofilm Coverage of the MPs Surface

The surface area of MPs covered by biofilm differed significantly between treatments (GLM; *p* = 0.016; Table S5), but not between exposures (Table S5). However, *posthoc* tests did not reveal any differences in the area between experimental treatments and controls for either crayfish or fish across any of the passages (planned contrast; Table S6; Figure 2). The significant effect of treatment on biofilm coverage was attributed to the notable difference between control 2 and the crayfish treatment when all measurements from the first and second exposures were pooled together (planned contrast; *p* = 0.020; data not shown).

### 3.5 The Chemical Changes on the MPs Surface

**Figure 3:**
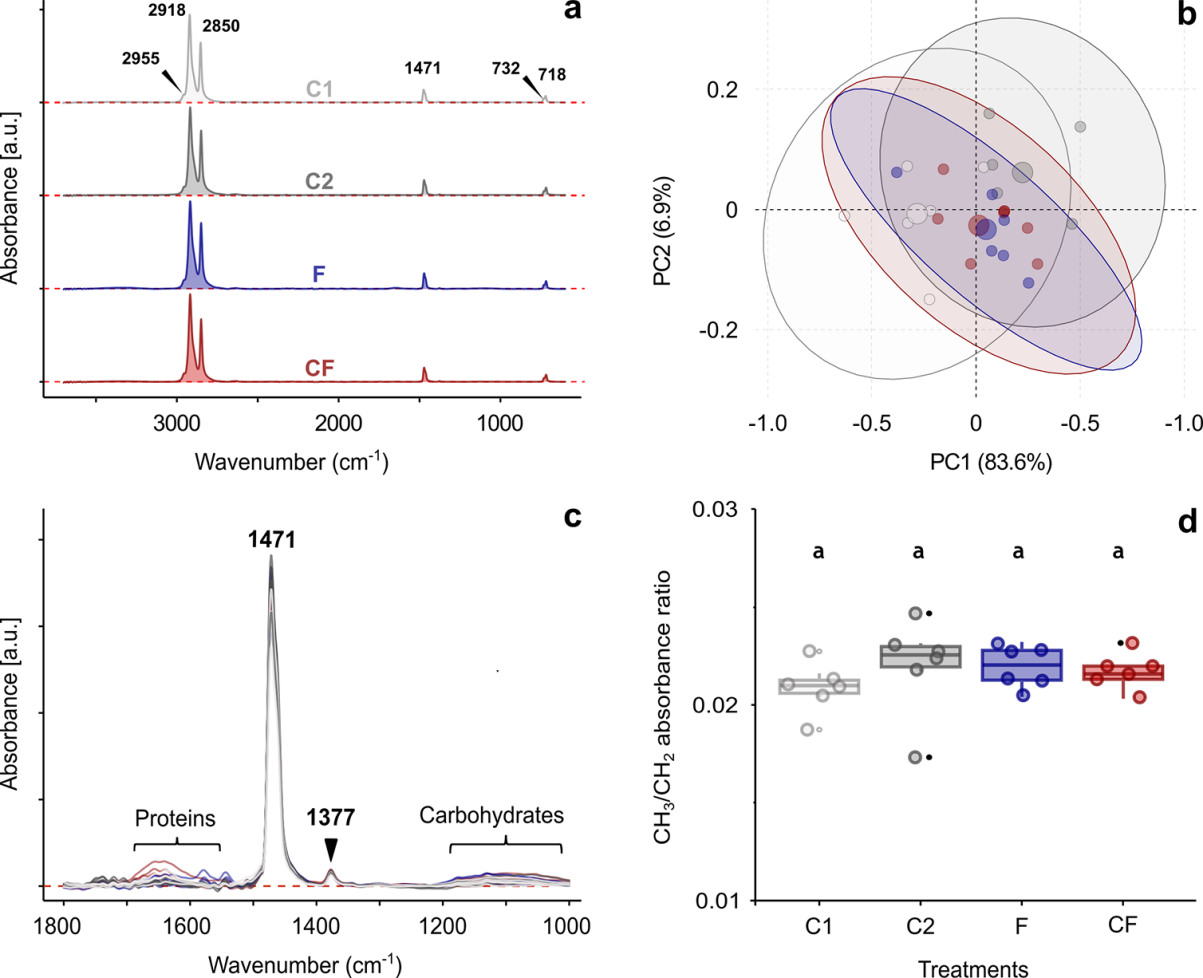
FTIR spectroscopy reflecting chemical compositionof the surface of MPs across different treatments after the second exposure of MPs: C1 (control 1), C2 (control 2), F (MPs after passage through the fish tract), and C (MPs after passage through the crayfish tract). (a) Mean spectra (*n* = 6) for each analyzed treatment. Peak positions of the most prominent bands are labeled. (b) PCA score plot for PC1 and PC2 based on the spectral data. The percentage of variance explained by these components is shown as axis labels. Ninety-five percent confidence ellipses are marked with different colors for each treatment level. Small points indicate sample scattering, while large points represent the average scores. (c) Magnification of the spectra within the wavenumber region between 1800 and 1000 cm^–1^. (d) Boxplot presenting the ratios of absorbance at 2955 and 2918 cm^–1^ calculated from the infrared spectra of MPs for control 1 and control 2, as well as for the double passage through the digestive tracts of crayfish and fish, respectively. The *p*-values for *posthoc* comparisons between the controls (1 and 2) and both treatment levels (fish and crayfish) are depicted along with brackets. *P*-values greater than 0.05 are not considered statistically significant.

The infrared spectra collected from the surface of the MPs reflected, almost exclusively, vibrations of the methylene (CH_2_) groups manifested at different modes (Figure 3a). Additionally, a weak shoulder at 2955 cm^–1^, assigned to the asymmetric CH_3_ stretching, and at 1377 cm^–1^ (the CH_3_ umbrella mode) were also resolved, indicating the presence of terminal methyl groups and possible branching.^52^ The most prominent peak, located at 2918 cm^–1^, corresponded to asymmetric stretching of the methylene groups. It was chosen as a reference peak for spectra normalization. In turn, symmetric stretching vibrations of CH_2_ were observed at 2850 cm^–1^. Much weaker, medium bands were related to CH_2_ bending (1471 cm^–1^) and CH_2_ rocking (732 and 718 cm^–1^) vibrations. We would like to emphasize that the splitting of the CH_2_ rocking band (Figure 3a) indicates the presence of crystalline regions in the examined PE samples.^53,54^ To evaluate possible chemical modifications of the MPs as a result of the treatments in more detail, we performed multivariate analysis, namely Principal Component Analysis. This unsupervised machine learning algorithm did not show any homogeneous subgroups for the first two principal components (PC1 and PC2), which together explained about 90% of the variability (Figure 3b). Similarly, overlapping of the PCA scores for the treatments and controls was observed for PC3 and PC4 (data not shown). Inspection of the spectra at higher magnification (Figure 3c) showed the presence of very weak bands in the regions of proteins (Amide I and Amide II) and carbohydrates, indicating some residues of organic matter. The ratio of peaks (area) at 2955 and 2918 cm^–1^, corresponding to the ratio of methyl to methylene groups, also did not change compared to control (ANODEV, χ^2^ (df = 3) = 3.03; *p* = 0.386) (Figure 3d).

### 3.6 The Density of Bacteria on the Surface of MPs

**Figure 4:**
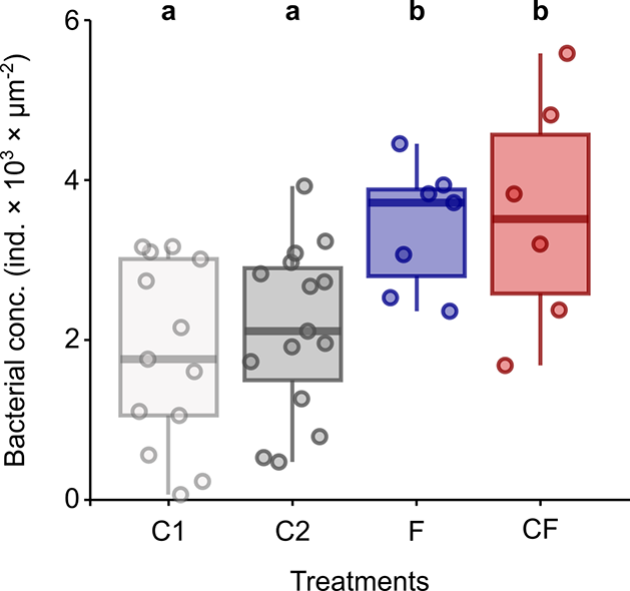
Density of bacteria on the surface of MPs across different treatments after the second exposure to MPs: C1 (control 1), C2 (control 2), F (MPs after passage through the fish tract), and C (MPs after passage through the crayfish tract). The vertical line in the boxplots represents the median, and the interquartile range indicates 50% of the data. The top and bottom whiskers correspond to the 75th and 25th percentiles, respectively. Significant differences between the comparisons (Table S8) are indicated by different Latin letters.

The density of bacteria on the surface of MPs was analyzed for the second exposure only, and the results revealed that the density differed between treatments (LM; *p* = 0.003; Table S7). More specifically, the density of bacteria on the MPs surface was higher after consumption by fish compared to both the first and second controls (planned contrast; *p* = 0.021 and *p* = 0.050, respectively; Table S8; Figure 4). Additionally, the passage of MPs through the digestive tract of crayfish resulted in a higher density of bacteria on their surface compared to both controls (planned contrast; *p* = 0.017 and *p* = 0.045 for the first and second controls, respectively; Table S8; Figure 4). No significant differences in bacterial density were observed between the two controls or between fish and crayfish (Table S8; Figure 4).

## 4 Discussion

In line with our first hypothesis, we found that the passage of MPs through the digestive tracts of crucian carp and Australian redclaw crayfish increases the damaged surface area of these particles. Within a short time frame (approximately 24 h), ingestion significantly alters the surface properties of MPs and reduces their size. The ingested MPs undergo mechanical breakdown, and the chipped fragments either penetrate the gut tissues or are excreted into the environment. Although we did not quantify the excreted chipped particles in our study, we can assume that they significantly outnumbered the particles that penetrated the gut tissues, as we did not detect any traces of PE in the gut tissues or glands of either fish or crayfish. The negligible penetration of NPs into the tissues did not confirm the predictions of our first hypothesis and contradicts the findings of previous studies [e.g.,^23,55^], which was most likely due to the short exposure time to MPs (limited to only two passages through the digestive tract) in our study compared to the earlier studies. Ultimately, the undigested fragments are excreted as smaller particles into the environment, although we did not quantify these particles in our study. While previous research has explored the effects of several groups of animals, mainly benthic and pelagic marine and freshwater crustaceans, on the qualitative and quantitative characteristics of MPs,^23−26,56^ our study is the first to specifically demonstrate this effect in crayfish and fish. Furthermore, only one prior study has examined the role of freshwater animals in the fragmentation of MPs.^25^ The results for fish are particularly significant, given their crucial role in the functioning of both marine and freshwater ecosystems.

It is essential to note that neither our study nor previous research has estimated the impact of plastic fragmentation by animals on the concentration of MPs in the environment. On one hand, animals can break down macroplastics and mesoplastics into MPs,^57−61^ and on the other hand, they can further degrade MPs into NPs.^23,25,55^ Currently, there is no research indicating whether animal activity has a greater influence on the first or second process. Investigating whether the foraging activity of animals increases or decreases the concentration of MPs and NPs in the environment presents an interesting avenue for future research.

Regardless of how animal activity affects the concentration of MPs, the number of plastic particles increases while their size decreases after passage through the digestive system. This makes them more readily ingested by organisms across various trophic levels compared to larger pieces.^2,61−65^ The increased relative density of smaller particles enhances their biofouling potential due to the rise in the surface area-to-volume ratio of the fragmented plastic, resulting in a higher likelihood of ingestion by smaller organisms further down the water column.^66,67^ Furthermore, this increase in the surface area-to-volume ratio, resulting from both size reduction and increased surface roughness of the particles, enhances the area available for microbial colonization, potentially accelerating their degradation.^68,69^ Additionally, the increase in the surface area-to-volume ratio of MPs may enhance their chemical harmfulness [e.g.,^70−74^] Finally, size-reduced particles are known to biodegrade more rapidly, indicating that smaller MPs have a shorter half-life in the environment.^75^ All of these factors suggest that the mechanical fragmentation of MPs by animals plays a significant role in altering both the abundance and distribution of MPs, as well as shaping their size structure. Consequently, this process may affect their availability and harmfulness to organisms.

In our study, we focused on a single fish species and one crayfish species. However, research indicates that the fragmentation process in the digestive tract varies depending on the foraging and feeding behaviors of different species, even among closely related ones.^61^ Furthermore, we examined only pristine MPs of a specific type, shape, and size. Studies suggest that the production of plastic fragments differs based on the condition of the plastics.^17,57,58,76^ For instance, the extent of defects in MPs largely depends on the type of polymer being tested. LDPE used in our study exhibits relatively high resistance to mechanical damage due to its branched structure and densely packed polymer chains.^77^ While the molecular weight of LDPE significantly affects its durability against environmental degradation, with higher molecular weights providing increased resistance to degradation processes, it is more susceptible to fragmentation than HDPE due to its lower crystallinity and more flexible structure. In contrast, PS, another commonly found plastic in the environment, is more brittle and less resistant to chemical degradation,^78,79^ making it more prone to damage in digestive systems.^76^ Furthermore, the fragmentation rate in animals can also depend on the sizes and shapes of ingested particles, as these features play an important role in their residence time within the digestive system and the ratio of their surface area to volume.^17^ The changes in MPs might have been more pronounced if a different type of plastic, such as fibers, had been used. Fibers are more likely to become lodged in the digestive tract and remain in the animal’s system longer.^80^ Another factor that can affect the rate of fragmentation is the fouled surface of MPs, which promotes fragmentation.^57,58^ Given the differences described above, future studies should expand to include the varying impacts of different fish and crayfish species, as well as the effects of MPs with diverse characteristics, to obtain a more comprehensive understanding of the role of these organisms in the mechanical fragmentation of MPs.

The first and second passages of MPs through the digestive tracts of fish and crayfish did not increase biofilm formation on the surfaces of the MPs, contrary to our second hypothesis. This lack of significant difference may result from two opposing processes: enzymatic activity during digestion could reduce biofilm coverage, while structural changes on the egested MPs might enhance biofilm formation. It is also possible that, while the biofilm surface area did not increase, its thickness did. However, these are speculative explanations not supported by our current data. Our findings highlight the need for further research to understand the interactions between MPs, digestion, and biofilm formation in aquatic organisms.

Our third hypothesis was not supported, as we did not observe any changes in the chemical composition or molecular structure of the MPs’ surfaces, even after two passages. The spectral features resembled typical for PE^81−83^ and were consistent across MPs from both the control groups and those that had passed through the digestive tracts of fish and crayfish. Furthermore, the ratio of peaks at 2955 and 2918 cm^–1^, which corresponds to the ratio of methyl (−CH_3_) to methylene (−CH_2_) groups and can estimate molecular weight and branching of PE chains,^84^ showed no change, indicating that the average length and structure of the polymer chains remained unaltered. This contrasts with findings from studies on terrestrial invertebrates,^17^ that reported chemical changes in ingested plastics. The shorter exposure time to MPs in our study (approximately 24 h), compared to longer exposure times in terrestrial studies,^85−87^ may explain this discrepancy. Additionally, the crystallinity of the polymer structure, manifested as splitting of the methylene (−CH_2_) rocking band near 725 cm^–1^ into separate peaks at 732 and 718 cm^–1^,^53,54^ may explain the resistance of the MPs’ surface to chemical modifications. Longer retention or additional passages could potentially alter chemical structures, especially in more degradable plastics like PS compared to PE.^76^

The results of our study support the fourth hypothesis, as MPs that passed through the digestive tracts of fish and crayfish and were then exposed to media with a known concentration of free-living bacteria showed increased bacterial density on their surfaces. This is likely due to the increased surface roughness from scratches caused by mechanical damage during passage,^88^ as most bacteria were found in these scratches (Figure 1D). MPs with surface abrasions are more prone to bacterial colonization because these scratches create microenvironments that trap bacteria and organic matter, expanding the available surface area for microbial attachment and enhancing ion adsorption, which further promotes bacterial growth.^89^ Additionally, fecal matter lodged in these irregularities provides nutrients and protection for microbes.^90^ Over time, mechanical degradation can lead to enzymatic degradation as microbial activity increases. While we measured only bacterial density on the MPs’ surfaces, our previous research found that fish feeding can change the bacterial composition on MPs, favoring taxa that may degrade plastic.^69^ Bacterial colonization on MPs can also be influenced by several physicochemical properties, including the type of plastic [e.g.,^91−96^] The PE-MPs used in our experiments had a relatively rough surface compared to other pristine MPs (e.g., PS), suggesting that the use of such MPs may result in a less pronounced effect on bacterial colonization. On the other hand, PS is more susceptible to damage in digestive systems,^76^ which could lead to greater bacterial colonization compared to PE.

The results confirmed the predictions arising from the final hypothesis, as the second passage through the digestive tract, compared to the first, resulted in an increase in the surface area of MPs covered in scratches and a decrease in particle size in the case of crayfish. These findings align with previous research indicating that longer exposure of MPs to degrading factors accelerates their degradation.^15^

In conclusion, our study revealed a significant role for fish and crayfish in the mechanical degradation and microbial colonization of MPs, indicating that aquatic animals can influence the fate of plastics in the environment. However, we did not find evidence that passage through the digestive tracts of fish and crayfish contributes to biofilm formation or changes in the chemical structure of MPs made of PE. While fish and crayfish may have a relatively minor direct impact on the complete mineralization of MPs, they play a crucial role in the initial degradation process by fragmenting the MPs, altering particle surfaces, and facilitating their colonization by microorganisms.

Overall, our study advances the understanding of animal-induced fragmentation and bacterial colonization of MPs, demonstrating that aquatic animals play a role in shaping the fate of plastics in aquatic ecosystems. It underscores the importance of accounting for animal-induced fragmentation when modeling the sources and pathways of plastics in the environment. This perspective may help reconcile discrepancies between observed and predicted behaviors of MPs in aquatic environments,^97^ thereby informing more effective strategies to mitigate plastic pollution.

## Supporting Information Available

This file contains supplementary tables (S1−S8) presenting statistical analyses, including ANOVA and planned contrasts, related to microplastic volume, surface scratches, biofilm coverage, and bacterial density. It also includes Text S1 with R code used for statistical modeling and Figure S1 showing chromatograms from pyrolysis experiments. PDF
