# Supplementary material for: Microplastic Passage through the Fish and Crayfish Digestive Tract Alters Particle Surface Properties
Source: Environ Sci Technol. 2025 Mar 14;59(11):5693–703. doi: 10.1021/acs.est.4c08909 (PMC11948475; doi:10.1021/acs.est.4c08909)
Supplement: Supplementary file 1 — es4c08909_si_001.pdf [file es4c08909_si_001.pdf]

Supporting information

Microplastic passage through the fish and crayfish digestive tract alters particle surface properties

Ewa Babkiewicz[1,2*], Julita Nowakowska[3], Marcin L. Zebrowski[1], Selvaraj Kunijappan[4], Katarzyna Jarosińska[1], Rafał Maciaszek[5], Jacek Zebrowski[6], Krzysztof Jurek[7], Piotr Maszczyk[1]

[1] *Department of Hydrobiology, Institute of Functional Biology and Ecology, Faculty of Biology, University of Warsaw, Warsaw, Poland*
[2] *Biological and Chemical Research Centre, University of Warsaw, Warsaw, Poland*
[3] *Imaging Laboratory, Faculty of Biology, University of Warsaw, Warsaw, Poland*
[4] *Department of Biotechnology, Kalasalingam Academy of Research and Education, Krishnankoil, India*
[5] *Warsaw University of Life Sciences, Institute of Animal Science, Department of Animal Genetics and Conservation, Warsaw, Poland*
[6] *Institute of Biotechnology, College of Natural Sciences, ~~Poland,~~ University of Rzeszow, Rzeszow, Poland*
[7] *Faculty of Geology, Geophysics and Environmental Protection at the AGH University of Krakow, Poland*

*Correspondence: e-mail address: ewa.babkiewicz@cnbc.uw.edu.pl

The following is included as supporting information for this paper:
Number of pages: 7
Number of tables: 8
Number of figures: 1

Text S1. R code (R version 4.3.2) used to performed general linear models and generalized linear models analysis:

```
#Specify a column type to be factor or numeric
data$treatment<-as.factor(data$treatment)
data$exposure <-as.factor(data$exposure)
data$replication <-as.factor(data$replication)
data$volume<-as.numeric(data$volume)
data$bacteria<-as.numeric(data$bacteria)
data$scratches <-as.numeric(data$scratches)
data$volume<-as.numeric(data$volume)


#Analysis of MPs volume
#Linear model (package stats v. 3.6.2; R Core Team, 2024)
model.volume <- lm(volume ~ treatment*exposure, data)

#DHARMA residuals plot (package DHARMa v. 0.4.6; Hartig, 2024)
plot(DHARMa::simulateResiduals(model.volume))

#ANOVA based on LM (package car v. 3.1-3; Fox and Weisberg, 2019)
anova.volume <- Anova(model.volume)
View(anova.volume)

#Obtain estimated marginal means for linear and generalized linear models (package emmeans v. 1.10.5; Lenth, 2022)
emmeans.volume <- emmeans(model.volume, ~ treatment:exposure)

#Pairwise comparisons of the estimated marginal means with Holm's adjustment for multiple testing (package emmeans v. 1.10.5; Lenth, 2024)
contrasts.volume <- pairs(emmeans.volume, adjust=”holm”)
View(contrasts.volume)


#Analysis of bacteria density on the surface of MPs
#Linear model (package stats v. 3.6.2; R Core Team, 2024)
model.bacteria <- lm(bacteria ~ treatment, data)

#DHARMA residuals plot (package DHARMa v. 0.4.6; Hartig, 2024)
plot(DHARMa::simulateResiduals(model.bacteria))

#ANOVA based on LM (package car v. 3.1-3; Fox and Weisberg, 2019)
anova.bacteria <- Anova(model.bacteria)
View(anova.bacteria)

#Obtain estimated marginal means for linear and generalized linear models (package emmeans v. 1.10.5; Lenth, 2022)
emmeans.bacteria <- emmeans(model.bacteria, ~tr)

#Pairwise comparisons of the estimated marginal means with Holm's adjustment for multiple testing (package emmeans v. 1.10.5; Lenth, 2024)
contrasts.bacteria <- pairs(emmeans.bacteria, adjust=”holm”)
```

```
View(contrasts.bacteria)


#Analysis of percentage of MPs covered by scratches
#Generalised linear model (package glmmTMB v. 1.1.10; Brooks et al., 2017)
model.scratches <- glmmTMB(scratches ~ treatment*exposure, family=beta_family(link = "logit"), data)

#DHARMA residuals plot (package DHARMa v. 0.4.6; Hartig, 2024)
plot(DHARMa::simulateResiduals(model.scratches))

#ANOVA based on GLM (package car v. 3.1-3; Fox and Weisberg, 2019)
anova.scratches <- Anova(model.scratches)
View(anova.scratches)

#Obtain estimated marginal means for linear and generalized linear models (package emmeans v. 1.10.5;
Lenth, 2022)
emmeans.scratches <- emmeans(model.scratches, ~ treatment:exposure)

#Pairwise comparisons of the estimated marginal means with Holm's adjustment for multiple testing
(package emmeans v. 1.10.5; Lenth, 2024)
contrasts.scratches <- pairs(emmeans.scratches, adjust=”holm”)
View(contrasts.scratches)


#Analysis of percentage of MPs biofilm surface coverage
#Generalised linear model (package glmmTMB v. 1.1.10; Brooks et al., 2017)
model.biofilm <- glmmTMB(biofilm ~ treatment*exposure, family=beta_family(link = "logit"), data)

#DHARMA residuals plot (package DHARMa v. 0.4.6; Hartig, 2024)
plot(DHARMa::simulateResiduals(model.biofilm))

#ANOVA based on GLM (package car v. 3.1-3; Fox and Weisberg, 2019)
anova.biofilm <- Anova(model.biofilm)
View(anova.biofilm)

#Obtain estimated marginal means for linear and generalized linear models (package emmeans v. 1.10.5;
Lenth, 2022)
emmeans.biofilm <- emmeans(model.biofilm, ~ treatment:exposure)

#Pairwise comparisons of the estimated marginal means with Holm's adjustment for multiple testing
(package emmeans v. 1.10.5; Lenth, 2024)
contrasts.biofilm <- pairs(emmeans.biofilm, adjust=”holm”)
View(contrasts.biofilm)
```

References

Brooks, M. E., Kristensen, K., Van Benthem, K. J., Magnusson, A., Berg, C. W., Nielsen, A., ... & Bolker, B. M. (2017). glmmTMB balances speed and flexibility among packages for zero-inflated generalized linear mixed modeling. *The R journal*, *9*(2), 378-400.

Fox, J., Weisberg, S., Adler, D., Bates, D., Baud-Bovy, G., Ellison, S., ... & Heiberger, R. (2012). Package ‘car’. *Vienna: R Foundation for Statistical Computing*, *16*(332), 333.

Hartig, F. 2024. _DHARMa: Residual Diagnostics for Hierarchical (Multi-Level / Mixed) Regression Models_. R package version 0.4.7, <https://CRAN.R-project.org/package=DHARMa>.

Lenth, R. 2024. _emmeans: Estimated Marginal Means, aka Least-Squares Means_. R package version 1.10.6, <https://CRAN.R-project.org/package=emmeans>.
R Core Team. 2024. _R: A Language and Environment for Statistical Computing_. R Foundation for Statistical Computing, Vienna, Austria. <https://www.R-project.org/>.

Table S1. Analysis of variance table from the linear model was conducted to test the effects of treatment (T), exposure (E), and their interaction on the volume of MPs. Statistically significant differences are indicated in bold (SS – sum of squares, df – degrees of freedom, F – F-statistic, *p* – *p*-value associated with the F-statistic).

Factors; Interaction	SS	df	F	*p*
T	**4.44E+13**	**3**	**40.727**	**<0.001**
E	**3.54E+12**	**1**	**9.746**	**0.003**
T × E	2.02E+12	3	1.856	0.145
Res.	2.51E+13	69		

Table S2. Planned contrasts for estimated marginal means using a linear model to assess the differences in the volume of MPs between treatments, which are combinations of two variables: treatments (C1 – MPs in experimental media (control 1), C2 – MPs suspended in food without passage through the digestive tract (control 2), F – MPs after passage through the fish tract, C – MPs after passage through the crayfish tract) and exposures (E1 – first exposure of MPs, E2 – second exposure of MPs). Statistically significant differences are shown in bold (E – estimate, SE – standard error, T – T-statistic, *p* – *p*-value).

Contrasts	E	SE	T	*p*
C1 × E1 – C2 × E1	-2.59E+05	3.01E+05	-0.859	1.000
C1 × E1 – F × E1	**1.59E+06**	**2.75E+05**	**5.768**	**<0.001**
C1 × E1 – C × E1	7.52E+05	2.80E+05	2.682	0.057
C2 × E1 – F × E1	**1.85E+06**	**2.75E+05**	**6.709**	**<0.001**
C2 × E1 – C × E1	**1.01E+06**	**2.80E+05**	**3.607**	**0.006**
F × E1 – C × E1	**-8.36E+05**	**2.52E+05**	**-3.321**	**0.011**
C1 × E2 – C2 × E2	1.61E+04	2.93E+05	0.055	1.000
C1 × E2 – F × E2	**1.71E+06**	**2.66E+05**	**6.442**	**<0.001**
C1 × E2 – C × E2	**1.59E+06**	**2.84E+05**	**5.611**	**<0.001**
C2 × E2 – F × E2	**1.70E+06**	**2.75E+05**	**6.165**	**<0.001**
C2 × E2 – C × E2	**1.58E+06**	**2.93E+05**	**5.389**	**<0.001**
F × E2 – C × E2	-1.18E+05	2.66E+05	-0.443	1.000
C1 × E1 – C1 × E2	1.16E+05	2.93E+05	0.395	1.000
C2 × E1 – C2 × E2	3.91E+05	3.01E+05	1.296	1.000
F × E1 – F × E2	2.41E+05	2.46E+05	0.979	1.000
C × E1 – C × E2	**9.59E+05**	**2.71E+05**	**3.538**	**0.007**

Table S3. Analysis of deviance from the generalized linear model with a beta distribution was conducted to test the effects of treatment (T), exposure (E), and their interaction on the surface area of MPs covered by scratches. Statistically significant differences are indicated in bold (χ^2 - chi-squared statistic, df - degrees of freedom, *p* - *p*-value associated with χ^2).

Factors; Interactions	df	χ2	*p*
T	**3**	**46.774**	**<0.001**
E	**1**	**5.479**	**0.019**
T × E	**3**	**8.056**	**0.045**

Table S4. Planned contrasts for estimated marginal means using a generalized linear model with a beta distribution to assess the differences in the surface area of MPs covered by scratches between treatments, which are combinations of two variables: treatments (C1 – MPs in experimental media (control 1), C2 – MPs suspended in food without passage through the digestive tract (control 2), F – MPs after passage through the fish tract, C – MPs after passage through the crayfish tract) and exposures (E1 – first exposure of MPs, E2 – second exposure of MPs). Statistically significant differences are shown in bold (E – estimate, SE – standard error, T – T-statistic, *p* – *p*-value).

Contrasts	E	SE	T	*p*
C1 × E1 – C2 × E1	-0.218	0.511	-0.426	1.000
C1 × E1 – F × E1	-1.231	0.462	-2.664	0.085
C1 × E1 – C × E1	-1.044	0.505	-2.070	0.308
C2 × E1 – F × E1	-1.013	0.460	-2.202	0.277
C2 × E1 – C × E1	-0.827	0.503	-1.643	0.702
F × E1 – C × E1	0.186	0.447	0.417	1.000
C1 × E2 – C2 × E2	-0.640	0.485	-1.321	1.000
C1 × E2 – F × E2	**-1.979**	**0.433**	**-4.566**	**<0.001**
C1 × E2 – C × E2	**-2.829**	**0.447**	**-6.330**	**<0.001**
C2 × E2 – F × E2	**-1.338**	**0.455**	**-2.939**	**0.040**
C2 × E2 – C × E2	**-2.189**	**0.466**	**-4.697**	**<0.001**
F × E2 – C × E2	-0.851	0.387	-2.196	0.277
C1 × E1 – C1 × E2	0.250	0.486	0.514	1.000
C2 × E1 – C2 × E2	-0.173	0.509	-0.339	1.000
F × E1 – F × E2	-0.498	0.390	-1.276	1.000
C × E1 – C × E2	**-1.535**	**0.450**	**-3.413**	**0.008**

Table S5. Analysis of deviance from generalised linear model with beta distribution to test the effect the treatment (T) and exposure (E) and their interaction on the biofilm coverage of MPs surface. Statistically significant differences are shown in bold (χ^2 - chi-squared statistic, df- degrees of freedom, *p* – *p*-value associated with a χ^2).

Factors; Interactions	df	χ2	*p*
T	**3**	**10.320**	**0.016**
E	1	0.033	0.855
T × E	3	1.460	0.691

Table S6. Planned contrasts for estimated marginal means using a generalized linear model with a beta distribution to assess the differences in the surface area of MPs covered by biofilm between treatments, which are combinations of two variables: treatments (C1 – MPs in experimental media (control 1), C2 – MPs suspended in food without passage through the digestive tract (control 2), F – MPs after passage through the fish tract, C – MPs after passage through the crayfish tract) and exposures (E1 – first exposure of MPs, E2 – second exposure of MPs). Statistically significant differences are shown in bold (E – estimate, SE – standard error, T – T-statistic, *p* – *p*-value).

Contrasts	E	SE	T	*p*
C1 × E1 – C2 × E1	0.663	0.451	1.470	1.000
C1 × E1 – F × E1	0.726	0.412	1.764	0.933
C1 × E1 – C × E1	0.967	0.456	2.120	0.510
C2 × E1 – F × E1	0.064	0.414	0.154	1.000
C2 × E1 – C × E1	0.305	0.458	0.665	1.000
F × E1 – C × E1	0.241	0.419	0.575	1.000
C1 × E2 – C2 × E2	0.118	0.424	0.278	1.000
C1 × E2 – F × E2	0.251	0.384	0.654	1.000
C1 × E2 – C × E2	0.962	0.402	2.393	0.268
C2 × E2 – F × E2	0.133	0.409	0.325	1.000
C2 × E2 – C × E2	0.845	0.426	1.981	0.666
F × E2 – C × E2	0.712	0.386	1.846	0.844
C1 × E1 – C1 × E2	0.230	0.424	0.542	1.000
C2 × E1 – C2 × E2	-0.315	0.450	-0.700	1.000
F × E1 – F × E2	-0.246	0.369	-0.666	1.000
C × E1 – C × E2	0.225	0.433	0.520	1.000

Table S7. Analysis of variance table from linear model, only for the second exposure, to test the effect of the treatment (T) on the density of bacteria on the surface of MPs. Statistically significant differences are shown in bold (SS – sum of squares, df – degree of freedom, F – F-statistic, *p* – *p* value associated with F-statistic).

Factors; Interactions	SS	df	F	*p*
T	**2.06E+07**	**3**	**5.563**	**0.003**
Res.	4.57E+07	37	-	-

Table S8. Planned contrasts for estimated marginal means using a linear model to assess the differences in the density of bacteria on the surface of MPs between treatments after the second passage of MPs: C1 – MPs in experimental media (control 1), C2 – MPs suspended in food without passage through the digestive tract (control 2), F – MPs after passage through the fish tract, C – MPs after passage through the crayfish tract). Statistically significant differences are shown in bold (E – estimate, SE – standard error, T – T-statistic, *p* – *p*-value).

Contrasts	E	SE	df	T	*p*
C1 – C2	-322.533	421.040	37	-0.766	0.897
C1 – F	**-1588.92**	**520.902**	**37**	**-3.050**	**0.021**
C1 – C	**-1755.49**	**548.392**	**37**	**-3.201**	**0.017**
C2 – F	**-1266.39**	**508.602**	**37**	**-2.490**	**0.050**
C2 – C	**-1432.96**	**536.723**	**37**	**-2.670**	**0.045**
F – C	-166.569	618.171	37	-0.269	0.897

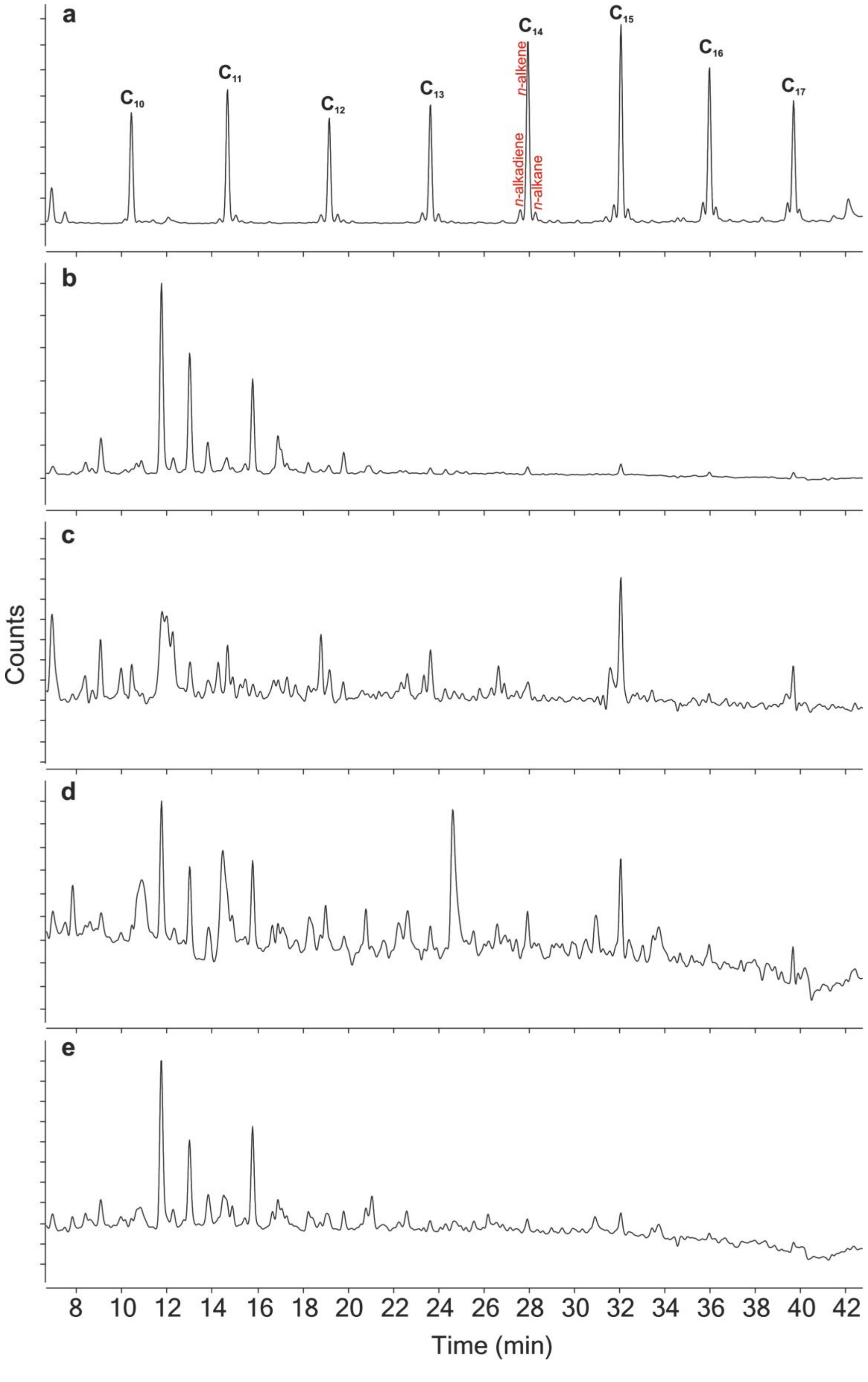

Fig. S1. Chromatogram for the standard showing the characteristic sequence of triplets for PE pyrolysis products (a), along with example chromatograms for the crayfish's hepatopancreas in the experimental sample (b) and control (c), and for the fish's intestine in the experimental sample (d) and control (e). The characteristic sequence of triplets for PE pyrolysis products was not detected in any of the control or experimental samples analyzed, indicating negligible penetration of PE-NPs into gut tissues and glands in both fish and crayfish.
